# Gait symmetry and regularity in transfemoral amputees assessed by trunk accelerations

**DOI:** 10.1186/1743-0003-7-4

**Published:** 2010-01-19

**Authors:** Andrea Tura, Michele Raggi, Laura Rocchi, Andrea G Cutti, Lorenzo Chiari

**Affiliations:** 1 Institute of Biomedical Engineering, National Research Council, Corso Stati Uniti 4, 35127 Padova, Italy; 2 Department of Electronics, Computer Science and Systems, University of Bologna, Viale Risorgimento 2, 40136 Bologna, Italy; 3 INAIL Prostheses Centre, Via Rabuina 14, 40054 Budrio (BO), Italy

## Abstract

**Background:**

The aim of this study was to evaluate a method based on a single accelerometer for the assessment of gait symmetry and regularity in subjects wearing lower limb prostheses.

**Methods:**

Ten transfemoral amputees and ten healthy control subjects were studied. For the purpose of this study, subjects wore a triaxial accelerometer on their thorax, and foot insoles. Subjects were asked to walk straight ahead for 70 m at their natural speed, and at a lower and faster speed. Indices of step and stride regularity (Ad1 and Ad2, respectively) were obtained by the autocorrelation coefficients computed from the three acceleration components. Step and stride durations were calculated from the plantar pressure data and were used to compute two reference indices (SI1 and SI2) for step and stride regularity.

**Results:**

Regression analysis showed that both Ad1 well correlates with SI1 (*R*^2 ^up to 0.74), and Ad2 well correlates with SI2 (*R*^2 ^up to 0.52). A ROC analysis showed that Ad1 and Ad2 has generally a good sensitivity and specificity in classifying amputee's walking trial, as having a normal or a pathologic step or stride regularity as defined by means of the reference indices SI1 and SI2. In particular, the antero-posterior component of Ad1 and the vertical component of Ad2 had a sensitivity of 90.6% and 87.2%, and a specificity of 92.3% and 81.8%, respectively.

**Conclusions:**

The use of a simple accelerometer, whose components can be analyzed by the autocorrelation function method, is adequate for the assessment of gait symmetry and regularity in transfemoral amputees.

## Background

Symmetry and regularity of walking are two important aspects in gait analysis. Symmetry is related to similarity of contralateral steps, whereas regularity is related to similarity of consecutive strides. Both symmetry and regularity of gait are usually impaired in subjects wearing lower limb prostheses [1-3]. The optimal use of a lower limb prosthesis is a challenging task, often requiring a long training for the amputee to achieve a nearly physiological pattern of movement [4-6]. In this context, a fundamental clinical issue is to verify whether the correct gait pattern learned during the physiotherapy sessions is maintained during autonomous walking. The presence or development of gait anomalies resulting in gait asymmetries [7] are known to be the cause of important comorbidities, such as low-back pain [8], osteoarthritis [9] and risk of falls [10], which can highly affect the quality of life of the subject. For these reasons, the restoration and persistence of a symmetric gait is one of the main targets in the rehabilitation of amputees.

In such a perspective, the availability of an easy-to-use, portable system capable of measuring the degree of gait symmetry and regularity may provide important contributions for the treatment of lower limb amputees, both in the clinical practice supporting professional caregivers (for reporting and decision-making in hospital or in out-clinics environment), and for home-care practice to support the patient for self-rating (for example in the home environment or during activities of daily life).

In this scenario, to facilitate the use of the system by both practitioners and patients, both in the hospital and in independent life, the device must implement the following features: low-cost, high-comfort, easy-mounting and low-maintenance requirements. For this purpose, the use of inertial sensors appears the most convenient choice, similarly to what has been done in other contexts, and only partially for lower-limb amputees [11-15], with only Robinson and colleagues [11] partially addressing the problem of gait symmetry and regularity.

From the on-board intelligence viewpoint, the development of a portable system for automatic detection of gait symmetry and regularity requires the selection of signal processing algorithms optimized for moderate processing resources consumption.

The aim of this study was therefore to assess the suitability of a method based on a single accelerometer and on the computation of the acceleration autocorrelation function [16], to measure the gait symmetry and regularity of unilateral transfemoral amputees (AMPs). For this purpose, we evaluated the correlation, sensitivity and specificity of the proposed approach with respect to reference indices computed from foot pressure measurements, together with their discriminative ability of detecting differences between AMPs and able-bodied subjects. To the best of our knowledge, this is the first study assessing gait regularity with inertial sensors in a group of transfemoral amputees.

## Methods

### Participants

Ten AMPs, all wearing a lower-limb prosthesis with the same kind of electronically controlled knee (C-leg, Otto-Bock, D) were recruited at the INAIL Prostheses Centre (Budrio, IT) for the study. All of them were confident walkers, since they had used mechanical prostheses for several years before using the electronically controlled knee, and by the time of measurements they had completed the training period with the C-leg. Ten healthy subjects were also studied as control group (CTRLs). Even if the control subjects resulted slightly younger than the amputees, they were, as the amputees, in the adult range of age, making the two groups suitable for the methodological validation of our approach. All participants were male and provided informed consent before data collection started. Further details on the two groups of subjects are presented in Table 1.

**Table 1:** Main characteristics of the two groups reported as means ± SE

	*AMP*	*CTRL*
*N*	10	10

*Age (years)*	45.7 ± 3.1	27.7 ± 1.2

*Height (m)*	175.9 ± 1.7	179.8 ± 1.5

Weight (kg)*	75.8 ± 2.2	73.4 ± 3.1

Walking velocity (km/h)**	4.0 ± 0.2	4.8 ± 0.3

*Cadence (steps/min)*	103.1 ± 2.5	113.8 ± 5.4

Prosthesis use duration (months)***	127.2 ± 38.0	/

*C-leg use duration (months)*	37.9 ± 10.5	/

* with prosthesis in AMP; ** at natural speed; *** from first fitting

### Equipment and set-up

Accelerometric data were acquired by means of an XSENS inertial sensing unit (MTx, XSENS Technologies B.V., NL). The sensing unit consists of a small case of 58 × 58 × 22 mm (WxLxH) weighing 50 g only. This includes some triaxial sensors: one accelerometer (full scale ± 50 m/s^2^), one gyroscope (full scale ± 300 deg/s) and one magnetometer, though in this study only the acceleration signals were considered. The sensing unit was placed on the thorax at the xiphoid process and fixed to the body through adhesive tape over an elastic bandage. Acceleration data were acquired with respect to the sensor's technical reference frame, which is certified by the manufacturer as being aligned along the MTx box borders with an error less than 3 degrees. The sensitive axes of the accelerometer were manually aligned along the anatomical vertical (V) axis (also named superior-inferior axis), and medio-lateral (ML) and antero-posterior (AP) axes. The sensing unit was connected to the XSENS data logger, which transmitted the data to a PC via Bluetooth.

To acquire the clinical reference measures, subjects also wore a pair of pressure insoles (Novel Gmbh, D) of proper size, based on capacitive sensor technology. Each insole provides up to 99 plantar pressure measurement spots. The Novel equipment was chosen since it is commonly used in the clinical practice, it has been widely validated in the literature [17,18] and it was previously used in the study of gait in subjects with amputations [19]. The acquisition of the pressure data was based on the Novel proprietary software PedarX. The two insoles were connected to the Novel data logger which stored the pressure data.

**Figure 1:**
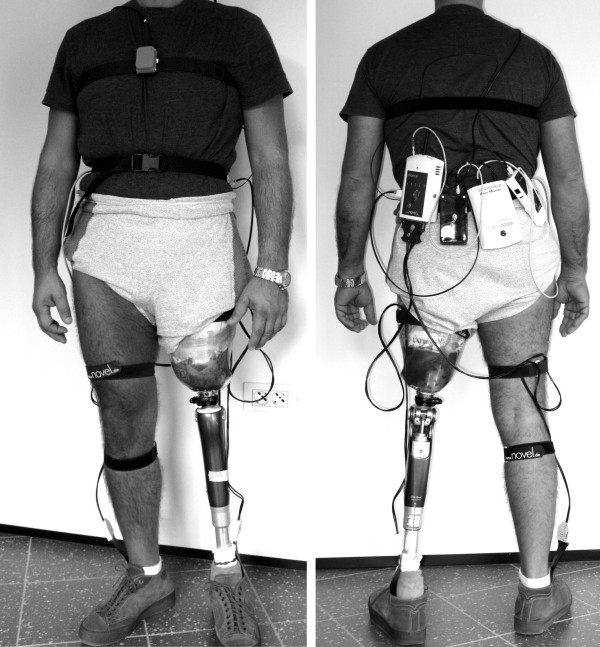
**The experimental set-up**. Front view (left) and rear view (right). The rear view shows the Novel data logger, the Novel battery, the XSENS data logger, and the Novel SyncBox (from left to right respectively).

A device was used to synchronize the acquisition from the XSENS and the Novel equipment (SyncBox, Novel Gmbh, D). The SyncBox was connected to the Novel data logger, and it received a clock signal from the XSENS data logger, that acted as master in the acquisition. A picture of the set-up is shown in Figure 1.

All the data were acquired at the sampling frequency of 100 Hz. Each MTx applied an anti-aliasing hardware filter (1^st ^order, cut-off frequency = 28 Hz) before digitalising the accelerometric signals. Data processing and analyses were performed in Matlab (The MathWorks Inc, US).

### Experimental protocol

Participants were asked to walk straight ahead along a hallway of the Prostheses Centre, for a total distance of 70 m. Firstly, subjects were asked to walk at their natural speed. Subsequently, the test was repeated and subjects were asked to walk at self-selected velocities, both slower and faster than their natural speed, with the following constraints: slow speed at least 20% lower than natural speed; fast speed at least 20% higher than natural speed. Compliance with these constraints was verified post-hoc by measuring the time taken by the subjects to walk the hallway. The analysis of gait at different speeds was aimed at reproducing the wide variability of walking conditions that may occur in the daily life. That allowed investigating a wide range of values in the symmetry and regularity indices, since velocity of walking may affect symmetry and regularity of gait [3]. The order of the tests was fixed (natural, slow, fast speed) and for each walking speed the test was repeated twice. Thus, a total of 6 gait tests were acquired for each subject, all containing at least 30 strides.

### Data analysis on accelerometric data

Gait symmetry and regularity indices were computed on the basis of the unbiased autocorrelation coefficients, according to the method proposed by Moe-Nilssen and Helbostad [16]. Briefly, the generic unbiased autocorrelation function of the sample sequence *x(i) *was computed by the following equation:

where *N *is the number of samples and *m *is the time lag expressed as number of samples.

We computed Ad(*m*) on each of the acceleration signals derived from the triaxial accelerometer during the gait tests. For each component we excluded from the analysis the samples related to the first and last five steps, to avoid transitional phases of gait initiation and termination.

The first peak of Ad(*m*), Ad1 coefficient, expresses the regularity of the acceleration between consecutive steps of the subject. This can be interpreted as a measure of the symmetry between steps performed by the prosthetic and the sound leg (or between left and right leg in CTRLs). The second peak of Ad(*m*), Ad2 coefficient, expresses the regularity of consecutive strides. Higher Ad1 (Ad2) values reflect higher step (stride) regularity. After normalization to the zero-lag component Ad(0) the maximum possible value for Ad1 and Ad2 is 1.

Values of Ad1 computed from the accelerometric signals along the vertical, medio-lateral and antero-posterior axes were indicated as Ad1_V_, Ad1_ML_, and Ad1_AP_, respectively. Similar nomenclature was used for Ad2, i.e. Ad2_V_, Ad2_ML_, and Ad2_AP_. Ad1 and Ad2 were identified within the autocorrelation function patterns through an automated procedure aimed at finding local maxima.

### Data analysis on pressure data

Plantar pressure data were analyzed through custom made software. The software computed the total vertical force at each time frame, deriving the time duration of each step and stride by detection of the time instants at which plantar pressure starts (heel-strike) or vanishes (toe-off). For each subject the force threshold indicating presence of foot contact was fixed to 10% of the mean vertical force maintained in orthostatic position for three seconds [20].

For each gait test, from the duration of steps and strides measured with the pressure insoles two reference indices of gait symmetry and regularity were calculated, firstly for each couple of consecutive steps and strides, and then averaged over the entire gait test.

For the regularity of steps (i.e. symmetry between legs) the following expression was used:

where T_STEP_R _and T_STEP_L _are the time duration of right and left step (from ipsilateral to contralateral heel-strike), respectively.

Similarly, for the regularity of strides the following expression was used:

where T_STRIDE_R _and T_STRIDE_L _are the time duration of a stride started with the right and left leg, respectively.

SI1(*i*) and SI2(*i*) were then averaged over the entire gait test to obtain the final values of SI1 and SI2:

where *N *(*M*) is the number of couples of steps (strides) in the gait test.

Such averaged values were assumed characteristics of the test and the corresponding standard deviations resulted negligible.

Although there is no unique index, in the scientific literature, accepted as reference for the computation of symmetry, expressions like SI1 and SI2 were widely used [21]. Also, SI1 and SI2 span the same range of possible values as Ad1 and Ad2, ranging from 0 to 1, the highest value representing complete gait symmetry/regularity. Thus, indices derived from pressure insoles were adopted as a valid reference method for the assessment of gait symmetry and regularity to be compared with accelerometer-based estimations.

### Statistical analyses

To validate the indices computed from the accelerometer through the autocorrelation analysis, the relation between Ad1 and SI1, and between Ad2 and SI2, were evaluated by means of univariate and multivariate regression analyses.

To see how well the symmetry and regularity indices could detect differences between AMPs and CTRLs, an ANOVA was carried out, with Repeated Measures to take into account the repeated tests for each subject, and with automatic corrections for violations of sphericity. A P value less than 0.05 was assumed for statistical significance. Results were reported as mean ± SE.

### ROC analysis

We performed a ROC analysis to measure the sensitivity and specificity of Ad1 (Ad2) in detecting a subject with "normal" or "pathologic" gait symmetry (regularity) during a test. For this purpose, a 5-step process was followed, here described for Ad1: 1) The SI1 values of all the tests in the CTRLs were displayed as a box & whisker plot; 2) Symmetry was assumed "normal" when the range of SI1 values was within the whiskers (1.5 times the interquartile range), and "pathologic" when outside the whiskers; 3) The SI1 values of all the tests in the AMPs were then considered, and each AMP's test was classified as featuring a "normal" or "pathologic" symmetry based on the previous definition: this was assumed as the reference classification for the AMPs' tests; 4) Each Ad1 value of the AMPs' tests was included in the "normal" or "pathologic" category according to the reference classification: we thus obtained two distributions of Ad1 values (for each of the three Ad1 indices); 5) Through ROC analysis on these two distributions, we determined the Ad1 threshold that maximises the correct classification of AMPs having "normal" or "pathologic" symmetry during a test.

Similar steps were performed with SI2 and Ad2 for stride regularity.

## Results

**Figure 2:**
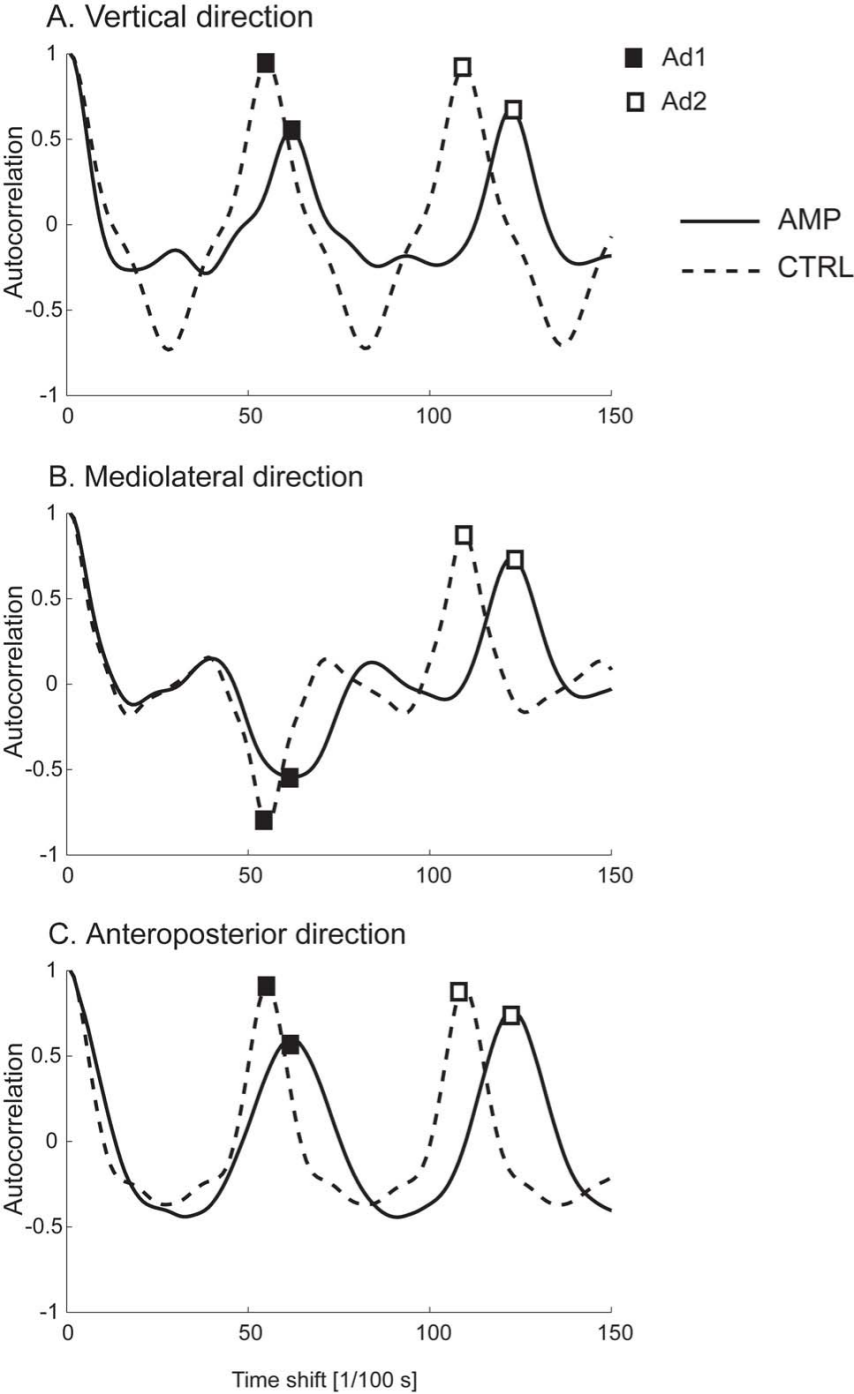
**Autocorrelation function computed during gait at natural speed**. Two representative subjects: amputee (solid line), control subject (dashed line). Ad1 and Ad2 values (peaks of the autocorrelation function) are indicated.

Representative patterns of the autocorrelation function computed from the three components of the acceleration signals in an AMP and in a CTRL are shown in Figure 2. As represented in the figure, Ad1_ML _values are always negative, both in AMP and in CTRL, since they correspond to the lateral trunk acceleration along the right-left directions (with opposite sign of the acceleration values when left stepping vs. right stepping). However, the absolute values were considered for the analyses. The patterns for the two subjects also show that the AMP's values for Ad1 and Ad2 are generally lower than CTRL's, for all the directions. Ad1 seems in general more different between the two subjects than Ad2.

**Figure 3:**
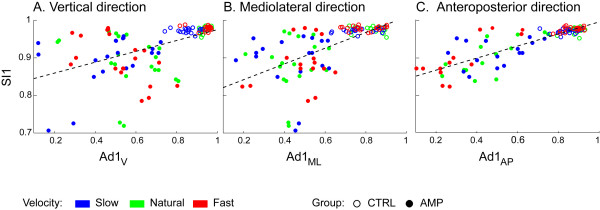
**Regression plots for Ad1_V_, Ad1_ML_, Ad1_AP _against SI1**. Solid circles: AMPs; empty circles: CTRLs. Blue, green, red symbols represent slow, natural, fast walks respectively. Regression related to all tests together is significant for each Ad1 index (*R*^2 ^= 0.285, *R*^2 ^= 0.398, *R*^2 ^= 0.735, respectively, P < 0.0001). Regression lines are Y = 0.83+0.14·X, Y = 0.80+0.21·X, Y = 0.84+0.16·X, respectively.

**Figure 4:**
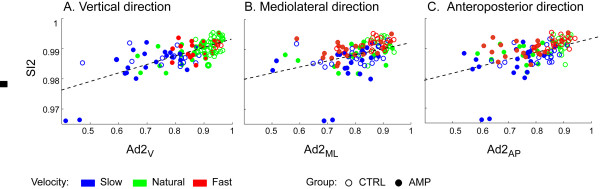
**Regression plots for Ad2_V_, Ad2_ML_, Ad2_AP _against SI2**. Solid circles: AMPs; empty circles: CTRLs. Blue, green, red symbols represent slow, natural, fast walks, respectively. Regression related to all tests together is significant for each Ad2 index (*R*^2 ^= 0.524, *R*^2 ^= 0.177, *R*^2 ^= 0.266, respectively, P < 0.0001). Regression lines are Y = 0.965+0.028·X, Y = 0.972+0.020·X, Y = 0.969+0.024·X, respectively.

Figures 3 and 4 report the results of the univariate regression analysis between accelerometry-based and pressure-based indices. When considering all the test sessions for all the subjects (AMPs+CTRLs) we found a good level of association between the indices. In particular, the highest correlations were found between SI1 and Ad1_AP _(*R*^2 ^= 0.735, P < 0.0001), and between SI2 and Ad2_V _(*R*^2 ^= 0.524, P < 0.0001). Therefore, any one of the three Ad1 indices may be considered a good surrogate of SI1 for the assessment of step regularity, and the same states for Ad2 indices for the assessment of stride regularity. Values of *R*^2 ^(and corresponding P) for all the indices are listed in captions of Figures 3 and 4.

Analysis of covariance showed that, for each index, there was no difference in the regression lines related to the three different walking speeds. Through the multivariate regression analysis, we found that any one of the three Ad1 indices contributes to explain the variability of SI1, i.e. all three indices were significant covariates (P < 0.016), with *R*^2 ^reaching the value of 0.776. As for Ad2 indices, in multivariate regression analysis only Ad2_V _was a significant covariate of SI2, whereas Ad2_ML _and Ad2_AP _were not.

Regression analyses were carried out, with AMPs and CTRLs treated separately in the analysis. In AMPs, no significant correlation was found between Ad1_V _and SI1, and the same for Ad1_ML_. Conversely, a significant correlation was found between Ad1_AP _and SI1 (*R*^2 ^= 0.401, P < 0.0001; regression line: Y = 0.83+0.17·X). Furthermore a significant correlation in SI2 was found with all the accelerometry-based indices, the best correlation being with Ad2_V _(*R*^2 ^= 0.570, P < 0.0001; regression line: Y = 0.960+0.035·X). Similarly, in CTRLs, no significant correlation was found between Ad1_V _or Ad1_ML _and SI1. For Ad1_AP _a significant though weak correlation was found with SI1 (*R*^2 ^= 0.127, P = 0.0052; regression line: Y = 0.93+0.05·X). Again, SI2 was significantly correlated with all the accelerometry-based indices, the best correlation being with Ad2_V _(*R*^2 ^= 0.326, P < 0.0001; regression line: Y = 0.974+0.019·X).

**Figure 5:**
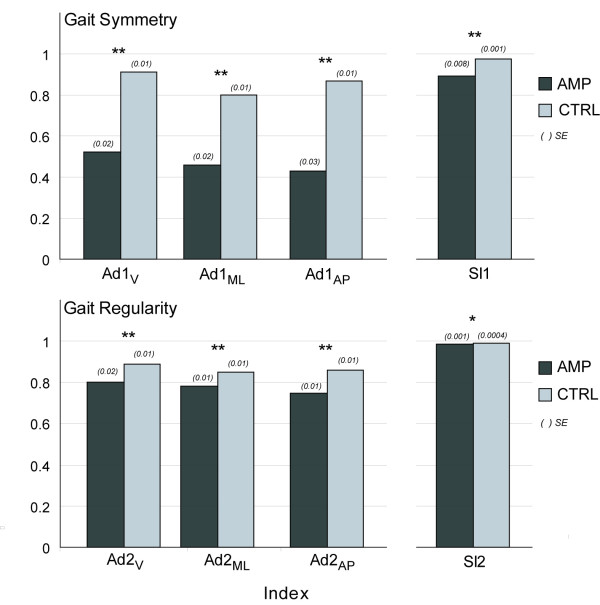
**Group comparisons of gait symmetry and regularity indices from thorax accelerometer and from pressure insoles**. Gait symmetry and regularity indices are Ad1_V_, Ad1_ML_, Ad1_AP_, Ad2_V_, Ad2_ML_, Ad2_AP_; pressure insoles indices are SI1 and SI2. Reported values are mean ± SE. All indices are non-dimensional. P-value of the differences in mean values of the two groups: *P = 0.0005; **P < 0.0001.

Mean values of all the indices in the two groups are shown in the bar graphs of Figure 5. As for the regularity of step (i.e. symmetry between consecutive steps), all the Ad1 indices, as well as SI1, were significantly different between AMPs and CTRLs (P < 0.0001). Similarly, in terms of regularity of stride, all the Ad2 indices (P < 0.0001), as well as SI2, (P = 0.0005) were different in the two groups.

**Figure 6:**
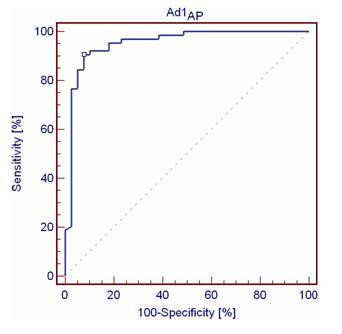
**ROC curve for Ad1_AP_**. Dot indicates the curve value at the highest accuracy.

By means of ROC analysis, two subjects among CTRLs were found having impaired walking tests in terms of gait symmetry assessed by SI1. Conversely, three subjects among AMPs had some normal walking tests. As for gait regularity assessed by SI2, only one CTRL had one impaired walking test, whereas all the AMPs had one or more normal walking tests. Table 2 reports the results of the sensitivity and specificity analysis of the various Ad1 and Ad2 indices. An exemplary ROC plot is shown in Figure 6. It can be noted that indices related to step had higher sensitivity and specificity than those related to stride.

**Table 2:** Sensitivity and specificity at the highest accuracy for Ad1 and Ad2 indices from ROC analysis

	*Cut-off value*	*Sensitivity (%)*	*Specificity (%)*	*AUC*_ROC _*
*Ad1*_*V*_	0.808	84.6	94.5	0.891

*Ad1*_*ML*_	0.6191	89.1	91.7	0.922

*Ad1*_*AP*_	0.7319	90.6	92.3	0.952

*Ad2*_*V*_	0.7666	87.2	81.8	0.919

*Ad2*_*ML*_	0.8164	61.5	90.9	0.784

*Ad2*_*AP*_	0.7688	73.4	100	0.866

* AUC_ROC _(range 0-1): area under the ROC curve

Since the time of use of the C-leg varied within a wide range (2 months to 7 years), the presence of a correlation between the duration of use and the gait performance was investigated, but no significant correlation was detected.

## Discussion

The aim of this study was to evaluate the appropriateness of a method based on the use of a single triaxial trunk accelerometer for the assessment of symmetry and regularity of gait in unilateral, transfemoral amputees. The interest for such measures is justified by their potential role in developing a portable automated device that may be able to evaluate the patient's gait features. In fact, one of the main characteristic that a portable, easy to use device to monitor gait features should have, is unobtrusive sensing units. Inertial sensors, such are accelerometers, are ideal candidate for such purpose. In this view, the methods and results presented in this study represent a step forward in the development of a potentially stand-alone portable system, that may act as a "virtual gait trainer" with the potentials of providing a summary score of walking ability in terms of gait symmetry and regularity during training, and, possibly, of alerting the therapist or the patient in the case of worsening of these gait features (biofeedback approach). The system could then be used even out of the clinics or rehabilitation institutes, allowing more frequent and prolonged training and rehabilitative therapy.

To these purposes, it was important to select a method for gait symmetry/regularity estimation that is particularly simple, both in terms of equipment and of computational requirements. In fact, if many approaches are possible for the estimation of gait regularity or gait variability [22,23], the main characteristic of the method based on the autocorrelation function proposed by Moe-Nilssen and Helbostad [16] is that it is extremely uncomplicated, thus adequate for possible implementation even on portable devices with limited computational resources (as a palmtop computer or a dedicated microprocessor-based unit, than can be worn by the user during unconstrained training of gait).

The proposed algorithm may provide information to the user regarding the *overall *gait performance, in terms of symmetry and regularity, since it requires a large number of consecutive steps to supply a reliable estimate of performance. This approach is indeed in accordance with the concept of task-oriented training, which has been recently confirmed as more appropriate than, e.g., single muscle or single body segment rehabilitation, when a specific motor function needs to be restored [24-26]. A device based on a single accelerometer is light, inexpensive, and easy to wear over the patient's clothes. On the contrary more established methods to estimate gait symmetry or regularity are often based on pressure insoles [27] or optical movement analysis systems [28]. Such systems are indeed reliable and widely described in the literature, but they are usually expensive, cumbersome, delicate in terms of maintenance, with a complex set-up, hence limited for a pervasive diffusion in the clinical practice or for home-based rehabilitation. In addition, Ad1 or Ad2 instead of temporal instants are preferable: they include information also on the morphology of the acceleration signals, not only on temporal features. Accelerometric data can potentially provide further information such as activity monitor functions and estimation of spatial parameters of gait. On the other side, systems based on pressure insoles have several drawbacks. In fact, the use of insoles is not comfortable for many patients, especially those using plantar supports, and a considerable amount of time may be necessary for some patients to wear them without help, as it may happen in the daily life; moreover, the insoles need to be of the specific patient's size; finally, systems based on insoles are usually very expensive and require an accurate calibration. Potential development of our approach toward a portable automatic device for gait training in subjects with lower limb prostheses will include further considerations, such as definition of the processing unit, identification of a simple and possibly wireless accelerometric unit, energy efficiency for long-lasting batteries. All these implementation issues are essential and will be arguments of further studies.

The analysis of gait symmetry and regularity in subjects wearing lower limb prostheses has been performed in previous studies. However, only few studies included healthy control subjects [1,2]. In [1], 11 unilateral transfemoral amputees and 2 control subjects were studied. The amputees were found to have an asymmetrical gait compared to control subjects, and the amount of asymmetry was related to the stump length. In [2], 9 unilateral transfemoral amputees and 18 control subjects were studied. The amputees showed asymmetry in their gait: for instance, the single support phase on the amputated side was shorter than on the intact side, whereas, as expected, no difference between the two sides was observed in control subjects. Our results are hence in agreement with both studies [1,2], since we found that gait indices computed from both the insole pressure measurement and from the accelerometer are lower in amputees than in control subjects.

As far as cross-validation of the two measurement systems is concerned, we found that Ad1 and Ad2 indices computed from the acceleration signals were well correlated with SI1 and SI2, hence the simple and inexpensive approach based on the use of a single accelerometer may be adequate to estimate gait symmetry and regularity in transfemoral amputees. To our knowledge, only a few studies used inertial sensors to evaluate gait in subjects with lower limb prosthesis [11-15], and only one of these studies addressed the issue of gait symmetry and regularity [11], but the study focused on below knee amputees and no control subjects were included. Of note, reliability of measures from accelerometers, in particular mounted on the trunk, was previously assessed with satisfactory results [29].

In the regression analysis, a possible limitation might be due to the inclusion of the data from all the repetitions for each subject. However, since the regression was performed on two measures both acquired during different tests, the independency between the samples remained despite the fact than more than one test resulted from the same subject.

As for the computation of Ad1 and Ad2 indices, comparison with other studies was possible only in relation to the control group. In [16], where the use of the autocorrelation function for gait analysis purposes was proposed for the first time, the authors found values for Ad1 and Ad2 very similar to the ones we estimated here (for instance, Ad1 = 0.89 and Ad2 = 0.91 from the vertical acceleration with a sensor at the L3 vertebra).

It is worth noting that the difference in Ad1 between AMPs and CTRLs was more marked than in Ad2 (absolute difference between the mean values equal to 0.27 and 0.10, respectively), and this is in agreement with what the physiotherapist expects. In fact, Ad1 represents the regularity of consecutive steps, and most likely in a subject wearing a unilateral prosthesis the right and left steps are different. Thus, it is reasonable that the differences compared with the CTRLs are more evident in the step regularity rather than in the stride regularity that may be still quite regular even in the amputees. As evidence, differences in SI2 between and AMPs and CTRLs were found statistically different, but appear clinically irrelevant.

In the analysis of AMPs and CTRLs grouped together, we found that each component of Ad1 (V, ML, AP) correlates with SI1, even if the degree of correlation (see *R*^2 ^values) differs between components. Similar results were found for Ad2 and SI2. However, no significant correlation was found between Ad1_V _or Ad1_ML _and SI1 in the single AMP and CTRL groups. In AMPs, that may indicate the existence of compensatory trunk asymmetry to regain some degree of gait symmetry, and that may reflect in relatively high SI1 values, differently to Ad1 values that are generally low. In CTRLs, a relation between Ad1 indices and SI1 may not be possible to demonstrate, because of lack of dispersion in the data. In all the subjects, it must also be noted that the correlation between Ad1 and SI1 (and similarly for Ad2 and SI2), even if significant, showed a slope of the regression line far from 1, i.e. they have much different range: SI1 and SI2 have in fact much narrower ranges compared to Ad indices. This was particularly observed in SI1 for the control subjects.

Even if there is not a standard reference method for the calculation of the symmetry indices [21] our results are robust to different formulation of the symmetry indices, since we tested some expressions (such as min(T_STEP_R_, T_STEP_L_)/mean(T_STEP_R_, T_STEP_L_) for the step, and similarly for the stride), and the main findings of the study were confirmed.

The sensitivity and specificity of Ad1 and Ad2 further support their use in the clinical practice. In particular, Ad1_AP _and Ad2_v _appear to be the best compromise between specificity and sensitivity for general uses, even though the 100% specificity for Ad2_AP _may be appealing when the amount of false positives is a major concern.

## Conclusions

We studied gait performance in a homogeneous group of prosthesis-aided patients, and we compared the symmetry and regularity of their gait with that of a population of control subjects. We found that a simple accelerometer, placed on the thorax at the xiphoid process may be adequate for the assessment of gait symmetry and regularity. Symmetry can be best assessed by the autocorrelation coefficient at the first dominant period computed from the acceleration along the anteroposterior axis (Ad1_AP_), and regularity by the coefficient at the second dominant period computed along the vertical axis (Ad2_v_). The use of the simple, low-cost accelerometry-based system will allow for early detection of asymmetric and irregular walking patterns; it will possibly be beneficial in the correction of these alterations to prevent related comorbidities, with potential wide penetration of this approach both in the clinical practice, and, on a future perspective, for home-based rehabilitation.

## Competing interests

The authors declare that they have no competing interests.

## Authors' contributions

AT has made substantial contributions to analysis and interpretation of data and has been involved in drafting the manuscript. MR has made substantial contributions to acquisition, analysis and interpretation of data. LR has made substantial contributions to analysis and interpretation of data and has been involved in revising the manuscript. AGC has made substantial contributions to conception and design, analysis and interpretation of data, and has been involved in revising the manuscript. LC has made substantial contributions to conception and design of the study and has been involved in revising the manuscript.

All authors read and approved the final manuscript.
